# Relationship between Peeled Internal Limiting Membrane Area and Anatomic Outcomes following Macular Hole Surgery: A Quantitative Analysis

**DOI:** 10.1155/2016/5641273

**Published:** 2016-06-16

**Authors:** Yasin Sakir Goker, Mustafa Koc, Kemal Yuksel, Ahmet Taylan Yazici, Abdulvahit Demir, Hasan Gunes, Yavuz Ozpinar

**Affiliations:** ^1^Ulucanlar Eye Training and Research Hospital, 06240 Ankara, Turkey; ^2^Beyoglu Eye Training and Research Hospital, 34420 Istanbul, Turkey; ^3^Kagıthane Government Hospital, Ophthalmology Department, 34416 Istanbul, Turkey

## Abstract

*Purpose*. To quantitatively evaluate the effects of peeled internal limiting membrane (ILM) area and anatomic outcomes following macular hole surgery using spectral domain optical coherence tomography (SD-OCT).* Methods*. Forty-one eyes in 37 consecutive patients with idiopathic, Gass stage 3-4 macular hole (MH) were enrolled in this retrospective comparative study. All patients were divided into 2 groups according to anatomic success or failure. Basal MH diameter, peeled ILM area, and MH height were calculated using SD-OCT. Other prognostic parameters, including age, stage, preoperative BCVA, and symptom duration were also assessed.* Results*. Thirty-two cases were classified as anatomic success, and 9 cases were classified as anatomic failure. Peeled ILM area was significantly wider and MH basal diameter was significantly less in the anatomic success group (*p* = 0.024 and 0.032, resp.). Other parameters did not demonstrate statistical significance.* Conclusion*. The findings of the present study show that the peeled ILM area can affect the anatomic outcomes of MH surgery.

## 1. Introduction

Internal limiting membrane (ILM) peeling is a crucial part of macular hole (MH) surgery [1], and using ILM peeling to remove and treat epiretinal membrane (ERM) improves anatomical outcomes [2]. Histological examinations show that ERM consists of pieces of the ILM [3]. The importance of the ILM in the pathogenesis of MH was also reported by Yoon et al. [4]. Pars plana vitrectomy (PPV) and ILM peeling are used to treat not only MH, but also ERM, diabetic macular edema, and retinal vein occlusion-related macular edema [5].

Optical coherence tomography (OCT) is the gold standard for diagnosing MH and assessing anatomic outcomes after surgery. OCT also provides prognostic information, such as basal MH diameter, MH height, MH minimum diameter, and other indexes of MH formation [6, 7]. Spectral domain optical coherence tomography (SD-OCT) can also assess structural changes in the macular layers, such as the inner and outer segment (IS/OS) and external limiting membrane [8, 9].

Age, basal MH diameter, MH index (MHI), stage, symptom duration, ILM peeling, and preoperative visual acuity affect the anatomic outcomes of MH surgery [10, 11]. However, no studies assess the relationship between peeled ILM area and anatomic outcomes following MH surgery. This study quantitatively evaluates the effects of peeled ILM area on open and surgically closed MHs.

## 2. Subjects and Methods

Forty-one eyes in 37 consecutive patients with idiopathic, Gass stage 3-4 MH were enrolled in this retrospective comparative study. The participants were classified as anatomic success or anatomic failure.

All MH cases underwent standard, sutureless, 3-port, 23-gauge vitrectomy surgery between March 2012 and March 2014. All surgeries were performed by the same surgeon (Ahmet Taylan Yazici) at Beyoglu Eye Research and Training Hospital. All patients received a complete ophthalmic examination, including measurement of best corrected visual acuity (BCVA) using an ETDRS chart, biomicroscopy of the anterior segment, and dilated fundus examination; all examinations were performed preoperatively on day 1 and week 1 and 1, 3, and 6 months after surgery. Spectral domain optical coherence tomography (SD-OCT) (SPECTRALIS® Heidelberg Engineering, Heidelberg, Germany) was used preoperatively to assess each patient and postoperatively at 1, 3, and 6 months.

Inclusion criteria were stage 3-4 MH according to the Gass classifications [12]. Exclusion criteria included refractive error > −6.00 D, traumatic MH, and history of ocular surgery (except phacoemulsification). Symptom duration was defined as the number of weeks from diagnosis to surgery. All patients provided informed consent prior to surgery, and this study adheres to the Declaration of Helsinki.

### 2.1. Surgery

All patients underwent standard, sutureless, 3-port, 23-gauge (G) pars plana vitrectomy (PPV) with triamcinolone acetonide- (TA-) assisted posterior vitreous detachment (PVD) (if not already present). The ILM was grasped using ILM forceps and peeled off the retina using 0.2 mL of brilliant blue G dye (Brilliant Peel; Geuder, Heidelberg, Germany). The area of the removed ILM was intended to reach the vascular arcades of the macula. Fluid-air exchange was performed through an extrusion cannula to flatten the hole, which was followed by the injection of 15% perfluoropropane (C_3_F_8_) or 20% sulfur hexafluoride (SF_6_). Patients were postoperatively maintained in the prone position for 5 days. Anatomic success was defined as complete MH closure and the absence of subretinal fluid on SD-OCT. Anatomic failure was defined as open MH after the first surgery.

### 2.2. SD-OCT

Every patient's SD-OCT parameters were separately analyzed by 2 observers. The initial set of measurements was recorded by the first observer. A second observer—who was blind to the results of the first observer—performed the same measurements in order to assess interobserver reproducibility. The first observer then scanned the same patient again to measure the same parameters and thereby assess intraobserver reliability. To reduce the likelihood of intraobserver bias, >10 minutes was allowed to elapse before the first observer repeated the measurements. The observers were not present in the OCT room during each other's examinations and were unaware of each other's final measurements.

**Figure 1:**
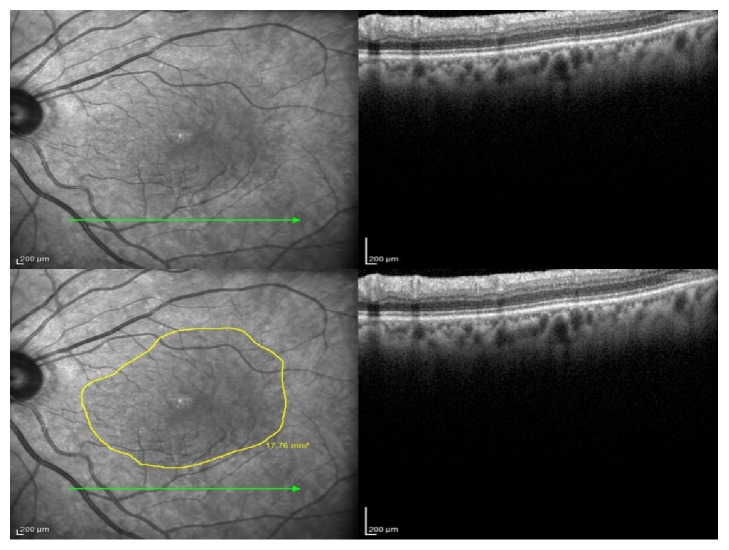
The borders of the peeled ILM area were marked, and the area was calculated using spectral domain optical coherence tomography.

Twenty-five horizontal scans through the fovea were preoperatively and postoperatively performed. Only the lowest section of the retinal macula was scanned to evaluate peeled ILM area. The borders of the peeled and nonpeeled ILM were seen and marked on the OCT scan. The software of the device calculates the area of the peeled ILM in square millimeters (Figure 1). The arithmetic means of by both observers were used in further analyses.

Basal MH diameter was defined as the diameter at the widest MH cross-section at the retinal pigment epithelium (RPE) [6, 7]. MH height was measured from the RPE to the top of the MH. MHI (hole height/basal hole diameter) was calculated using previously described methods [6]. Anatomic success was defined by complete MH closure and the absence of subretinal fluid on SD-OCT at month 1 postoperatively.

### 2.3. Statistical Analysis

The statistical analysis was performed using SPSS (Statistical Package for the Social Sciences) (version 16 for Windows; SPSS Inc.). The normality of the data was confirmed using the Kolmogorov-Smirnov test (*p* > 0.05). One-way ANOVA was used to evaluate homogeneity between groups (*p* > 0.05). Groups were analyzed using the parametric *t*-test or nonparametric Mann-Whitney test. Multiple regression analysis was used to determine if there was a significant association between anatomic outcomes and several factors, including Gass stage, basal MH diameter, peeled ILM area, MHI, symptom duration, and preoperative BCVA. BCVA was converted to logMAR (logarithm of the minimal angle of resolution) equivalents for the statistical analysis. In this study, *p* < 0.05 is considered statistically significant.

## 3. Results

Thirty-seven patients met our inclusion criteria, and 4 had bilateral MH. The mean follow-up period was 17.4 months (range = 6–30 months). Baseline parameters and patient demographic data are presented in Table 1. Thirty-two cases were included in the anatomic success group, and 9 cases were included in the anatomic failure group. The mean ages of the patients in each group were 67.1 ± 7.3 and 66.3 ± 5.7 years, respectively.

The clinical characteristics of participants are shown in Table 2. Thirty-four eyes were phakic, and 7 eyes were pseudophakic. Three patients developed significant cataracts during follow-up and underwent phacoemulsification with intraocular lens implantation. No significant difference in lens status was found between groups (*p* = 0.147). Phacoemulsification with intraocular lens implantation was combined with MH surgery in 2 cases. Therefore, combination surgery did not demonstrate a significant influence (*p* = 0.332). Perfluoropropane (C_3_F_8_) was used in 33 eyes as tamponade, and sulfur hexafluoride (SF_6_) was used in 8 eyes. There was no significant difference between eyes treated with C_3_F_8_ or SF_6_ in terms of anatomic outcomes (*p* = 0.616).

Mean preoperative BCVA was 0.85 ± 0.33 logMAR, which postoperatively improved to 0.66 ± 0.37 logMAR (*p* = 0.001) (0.87 ± 0.36 versus 0.80 ± 0.25 logMAR in patients classified as anatomic success or failure, resp., however, there was no significant difference between groups (*p* = 0.936)). Symptom duration was 18.9 ± 12.8 versus 17.22 ± 14.65 weeks in patients classified as anatomic success or failure, respectively. Therefore, symptom duration did not demonstrate a significant difference between groups (*p* = 0.738).

Mean peeled ILM area was 16.51 ± 6.15 mm^2^ (range = 3.90–30.0 mm^2^) and 12.8 ± 4.0 mm^2^ (range = 6.89–17.67 mm^2^) in patients classified as anatomic success or failure. There was a statistically significant difference between groups in terms of peeled ILM area (*p* = 0.024). Mean basal MH diameter was 963.2 ± 325.1 *μ*m (range = 302–1625 *μ*m) and 1426.0 ± 621.3 *μ*m (range = 760–2627 *μ*m) in anatomic success and failure patients, respectively. Basal MH diameter was also significantly different between groups (*p* = 0.032). Furthermore, there was a significant association between anatomic outcomes and 2 factors—basal MH diameter and peeled ILM area (*p* = 0.001 and 0.009, resp.)—according to the multiple regression analysis (shown in Table 3).

The primary and final anatomic success rates were 78% (32 of 41 cases) and 92.7% (38 of 41 cases), respectively. Overall, 9 cases remained open (anatomic failure) after the first surgery, and second surgery was recommended for 8 cases. One case that had not been recommended for second surgery developed wide RPE atrophy and would not have benefited from surgery. Among the open MHs, 2 patients could not postoperatively maintain the prone position for 5 days and subsequently refused additional surgery.

## 4. Discussion

Over the past 20 years, ILM peeling has played a crucial role in the surgical treatment of a variety of retinal disorders, including epiretinal membrane, MH, diabetic macular edema, and retinal vein occlusions. The available evidence supports using ILM peeling as the treatment of choice for patients with idiopathic stages 2–4 MH [13]. ILM removal relieves the forces around the fovea, including those that are tangential and axial; however, there is no general consensus regarding the extent of the ILM area that should be peeled [5]. In this retrospective study, we found that larger peeled areas demonstrated better anatomic outcomes.

Many factors affect anatomic outcomes, and age, Gass stage, basal MH diameter, MHI, preoperative BCVA, and symptom duration are some prognostic criteria for MH surgery [10, 11]. All could also affect anatomic outcomes. These parameters—including basal MH diameter, MHI, peeled ILM area, Gass stage, symptom duration, and preoperative BCVA—were assessed by our multiple regression analysis, but only MH basal diameter and peeled ILM area were found to be statistically significant.

Balducci et al. reported early and late changes in retinal nerve fiber layer thickness (RNFLT) after ILM peeling for idiopathic macular hole or epiretinal membrane [14]. RNFLT increased at 1 month after surgery, returned to preoperative levels by 3 months, and was lower than basal at 6 months after surgery. Balducci et al. proposed that reduced RNFLT at 6 months after surgery could indicate damage caused by ILM peeling. In addition, according to a retrospective study that used microperimetry, Tadayoni et al. reported that decreased retinal sensitivity was associated with paracentral absolute and relative microscotomas in 8 of 16 eyes following ILM peeling and MH surgery due to large macular holes (>400 mm) [15]. Some authors have proposed that ILM peeling causes the loss of Müller cell footplates and affects retinal function. Terasaki et al. reported delayed implicit time and reduced b-wave amplitude on focal electroretinography (ERG) soon after ILM peeling [16]. Steven et al. reported that ILM peeling may result in retinal weakening via Müller cell damage, which causes structural breakdowns and finally paracentral retinal hole formation. Steven et al., Mason III et al., and Rubinstein et al. have all separately reported the increased risk of secondary paracentral retinal hole formation after ILM peeling [17–19]. On the contrary, Che et al. evaluated 134 eyes in 130 IMH patients who received PPV in combination with ILM peeling (2 disk diameters). Thirteen eyes underwent a second surgery that involved enlarging the peeled ILM area to the vascular arcades of the posterior fundus. MH closure was successfully achieved in 8 of 13 eyes (61.5%) [20].

The surgeon may perform many manipulations to enlarge the peeled ILM area. The retina nerve fiber layers can hemorrhage and iatrogenic retinal holes may develop, and these hemorrhages may result in visual field defects and other retinal alterations. Accordingly, many surgeons do not widen the peeled area, and a smaller peeled ILM results in less of Müller cells loss, stronger retinal structure, lower risk of visual field defects, and paracentral retinal hole formation. On the other hand, small peeled ILM demonstrates worsened anatomic outcomes.

There is tangential traction in the etiology of macular hole formation that is induced by vitreous shrinkage, as observed and reported by Gaudric et al. [21]. We propose that wider ILM peeling relieves this traction more efficiently, therefore resulting in better anatomic outcomes. Here, the mean area of peeled ILM in anatomically successful patients was 16.51 mm^2^, whereas patients with anatomic failure demonstrated a mean area of 12.8 mm^2^. It is difficult to determine a good cut-off value for the peeled area that confirms the best anatomic outcomes. The surgeon should peel the ILM to as much close to the vascular arcades of the macula as possible.

The limitations of the present study include the relatively small numbers of patients, its retrospective design, and the fact that the peeled ILM borders were only assessed using SD-OCT. Therefore, the peeled area could have been inaccurately measured. Using preoperative ILM markings could improve ILM assessment. Also we did not histologically examine the peeled ILM. A strength of this study is the quantitative assessment of the peeled ILM using SD-OCT. In conclusion, we propose that peeled ILM area is important in MH surgery and that it can affect anatomic outcomes.

## Figures and Tables

**Table 1:** Baseline parameters and demographic data.

Variable	Anatomical success (Group 1)	Anatomical failure (Group 2)	*p* value
Eyes (*N*)	32	9	

Gender (*N*)			
Male	9 (31.0%)	2 (25%)	
Female	20 (69.0%)	6 (75%)	

Age, years			0.762^*∗*^
Mean ± SD	67.1 ± 7.3	66.3 ± 5.7	
Range	57–85	59–81	

Stage (*N*)			0.176^*∗∗*^
3	11 (34.4%)	1 (11.1%)	
4	21 (65.6%)	8 (88.9%)	

^*∗*^ *t*-test.

^*∗∗*^Mann-Whitney test.

**Table 2:** Clinical characteristics of participants.

Variable	Group 1	Group 2	*p* value
Preoperative BCVA, logMAR			0.936^*∗*^
Mean ± SD	0.87 ± 0.36	0.80 ± 0.25	
Range	0.4–1.8	0.52–1.3	

Symptom duration, weeks			0.738^*∗*^
Mean ± SD	18.9 ± 12.8	17.22 ± 14.65	
Range	4–64	4–40	

Lens status, *N*			0.147^*∗∗*^
Phakic	28 (87.5%)	6 (66.7%)	
Pseudophakic	4 (12.5%)	3 (33.3%)	

Tamponade, *N*			0.616^*∗∗*^
C_3_F_8_	27 (84.4%)	6 (66.7%)	
SF_6_	5 (15.6%)	3 (33.3%)	

Surgery, *N*			0.332^*∗∗*^
PPV	31 (96.9%)	8 (88.9%)	
Combined PPV + phaco	1 (3.1%)	1 (11.1%)	

MH basal diameter, *μ*m			0.032^*∗∗*^
Mean ± SD	963.2 ± 325.1	1426.0 ± 621.3	
Range	302–1625	760–2627	

MHI			0.347^*∗*^
Mean ± SD	0.53 ± 0.25	0.45 ± 0.10	
Range	0.28–1.55	0.30–0.68	

Peeled ILM area, mm^2^			0.024^*∗*^
Mean ± SD	16.51 ± 6.15	12.8 ± 4.0	
Range	3.90–30.0	6.89–17.67	

Bold values are significant at *p* < 0.05. BCVA, best corrected visual acuity; ILM, internal limiting membrane; MH, macular hole; MHI, macular hole index; PPV, pars plana vitrectomy; phaco, phacoemulsification; *μ*m, micrometer; mm^2^, millimeter square.

^*∗*^ *t*-test.

^*∗∗*^Mann-Whitney test.

**Table 3:** Multiple regression model of variables associated with anatomical outcome.

Variables	95% confidence intervals	*p* value
MH basal diameter	0.545–0.940	**0.001**
MHI	0.246–0.668	0.137
Peeled ILM area	0.111–0.467	**0.009**
Stage	0.335–0.409	0.461
Symptom duration	0.129–0.601	0.559
Preoperative BCVA	0.358–0.763	0.076

Bold values are significant at *p* < 0.01. BCVA, best corrected visual acuity; ILM, internal limiting membrane; MH, macular hole; MHI, macular hole index.
